# 

**Published:** 1862-11

**Authors:** 


### CHICAGO MEDICAL JOURNAL.

**Terms, $2.00 per Annum.**

### CONTENTS OF THE NOVEMBER NUMBER.

ORIGINAL COMMUNICATIONS.

PAGE.

Introductory Address Delivered at the Commencement of the Session of 1862–3 of Rush Medical College. By J. Adams Allen, M. D., LL. D., Professor of Principles and Practice of Medicine, and Clinical Medicine ........ 609

Extracts from a Letter from E. L. Holmes, M. D., of Chicago ........ 628

On the Difficulty of Always Making an Exact Diagnosis in Typhoid Fever. By G. C. Paoli, M. D., Chicago ........ 632

Malingering—No. 2. By Tom O. Edwards, M. D., Chicago ........ 637

SELECTED.

Causes of Failure in the Treatment of Uterine Ulcer. By Robert Ellis, Esq., Obstetric Surgeon to the Chelsea and Belgrave Dispensary, 648

The Turkish Bath in the Treatment of Insanity ........ 654

On Reproduction of Tendons whose Divided Ends have Separated over Four Inches. By Dr. Cooper ........ 655

Small-Pox.—Vegetarianism.—Early Marriages ........ 657

Editorial and Miscellaneous ........ 663

